# Intact social cognitive processes in outpatients with anorexia nervosa: a pilot study

**DOI:** 10.1186/s12991-016-0108-0

**Published:** 2016-09-01

**Authors:** Katarzyna Kucharska, Julia Jeschke, Reza Mafi

**Affiliations:** 1 Institute of Psychiatry and Neurology, 9 Sobieskiego Street, 02-957 Warsaw, Poland; 2 NAVIGO CIC, Grimsby, UK

**Keywords:** Social cognition, Emotion recognition, Facial perception, Empathy, Theory of mind

## Abstract

**Objective:**

The aim of the study was to assess social cognition in community patients suffering from anorexia nervosa (AN) compared to healthy controls.

**Methods:**

25 women diagnosed with AN and 25 women matched for education level and age were involved in the study. Both subject groups were assessed using a set of validated experimental tasks, such as the facial expression recognition test, short recognition memory test for faces, ‘Reading the mind in the eyes’ test. Patients were assessed for symptoms of: eating disorder (the eating attitudes test—EAT-26), OCD (the Yale–Brown obsessive compulsive scale—Y-BOCS) and depression (Beck depression inventory—BDI). The research hypothesis indicated that patients suffering from anorexia represent no significant difference in social cognitive functioning in comparison to the healthy controls. These assessment scales were used to identify whether there are any problems according to social cognitive functioning especially emotion recognition and theory of mind (ToM). The primary outcome assessment was to identify social cognitive deficits in anorexic outpatients and secondary outcome was to verify whether these problems in emotional functioning found in women in acute phase of AN are state or trait effects.

**Results:**

Anorexic patients showed significantly higher scores on EAT-26, BDI and Y-BOCS. No significant differences were found in performance of social cognitive tests and facial perception test.

**Discussion:**

No marked alterations were found in social cognitive functioning in community patients with average body mass index (BMI) of 17.6. This may indicate that social cognition is a very complex construct to be reliably measured in anorexia nervosa considering relatively limited psychometric data for many social cognitive tasks. Further longitudinal studies are needed to untangle ongoing controversy whether social cognitive deficits in AN could be state or trait related.

## Background

Anorexia nervosa (AN) is a severe mental disorder characterised by persistent restriction of food intake leading to significantly low body weight, intense fear of gaining weight or becoming fat or persistent behaviour that interferes with weight gain, and disturbance in the way in which one’s body weight or shape is experienced (DSM-5).

Adolphs [1] described social cognition as “the ability to construct representations of the relation between one-self and others and to use those representations flexibly to guide social behaviour”.

Social cognition has been defined as the mental operations underlying social interactions, and is thought to represent a specialised domain of cognition, which captures affect perception, social cue perception, “theory of mind”, empathy, and attribution style [18]. As far as facial affect recognition is concerned, robust research data seemed to report less accuracy of AN patients at recognising basic facial emotions [7, 16, 21, 33].

Furthermore, previous research has found that individuals with eating disorders exhibit lower emotional awareness compared to normal controls, impaired processes of theory of mind/mentalising, and empathy [2, 17, 20, 33]. Mentalisation plays crucial role in social cognition research and can be seen as a form of mental ability which allows to perceive and interpret human behaviour in terms of intentional mental states [10]. There have been suggestions that difficulties in socio-emotional aspects of anorexia nervosa presentation may be related to autistic type traits [13], and this leads to a hypothesis that impairments in theory of mind/mentalising might be seen [29]. For that reason participant groups were assessed on the ‘Reading the mind in the eyes’ task, which asked participants to identify a person’s mental state from photographs of people’s eyes.

In contrast, other studies have found no significant differences in decoding of emotional facial expressions in eating disordered individuals compared to healthy groups [2, 31], and any differences in levels of empathy compared to healthy controls groups [13]. Moreover, there is a growing body of literature showing intact social cognitive functioning in partially remitted patients with AN [23, 24]. However, considerable amount of research work indicates their presence to lesser extent and persistence despite remission of symptoms and normalisation of body weight [5].

Although not central to the diagnostic criteria of AN, emerging evidence suggests additionally deficits in key aspects of social functioning. Patients appear to be socially withdrawn, and they report having smaller social networks, less social interactions [19] and a reduced number of close friends [9].

Most empirical work has emphasised the role of eating in response to changes in affect; this research is focused on the possible role of social cognition in the maintenance of distorted eating [29]. In this particular study the hypothesis states that difficulties in negotiating the unpredictable and affect-laden social milieu may be contributing factor to maintenance of eating disorders.

Primary outcome measures include whether community patients with anorexia nervosa have specific deficits in social cognition per se or present with impaired processing of a more facial perceptual nature.

Social cognition in the anorexic patients was assessed using standardised measures in order to determine whether:

- the magnitudes of such impairments in patients were greater compared with healthy controls.
- the deficits of social cognition and facial processing were more “trait” rather than “state” dependent (correlational analyses with demographic and clinical factors).

State is a momentary emotional reaction to internal and/or external trigger(s) which also involves physical, behavioural, cognitive and psychological reactions. The duration and intensity of the emotion felt can vary due to various factors such as the level of arousal, frustration level, subjective perception, the context, etc. [30]. Traits refer to the stable, consistent and enduring disposition of the individual [3], which includes emotional reactions and temperament, rather than situational, variable and temporary factors [12].

## Methods

### Participants

This study was approved by the local ethics committee in Cardiff, the UK, and all participants gave informed consent.

Participants included 25 British females with anorexia nervosa, age range 16–45, (restricting and binge–purge type) fulfilling both International Classification of Diseases (ICD-10) and Diagnostic and Statistical Manual of Mental Disorders, 4th Edition, Text Revision (DSM IV TR) criteria were recruited from Eating Disorder Clinic, Cardiff and Vale NHS Trust and compared with 25 healthy participants recruited from students and non-medical staff within Cardiff and Vale NHS Trust (Table 1). Initially 28 patients were assessed from which 28 completed clinical measures alone and 25 completed both clinical and psychological measures. These drop-outs occurred due to a poor treatment compliance. Consensus diagnoses were obtained from senior clinicians who assessed the patients in clinical interviews. Inclusion criteria for control group were British healthy females, age range 16–45, with body mass index (BMI = weight in kilograms divided by height in meters squared) within normal range 18–26, correct hearing and vision. The exclusion criteria for the comparison group included symptoms of eating disorders, menstrual irregularity, pregnancy, a family history of an eating disorder, habitual drug or alcohol abuse, secondary neurologic disorders (e.g. epilepsy or dementia) and other psychiatric diagnoses. All the participants were native English speakers and were examined once. Patients were assessed at the beginning of the therapy (either at the second or third community appointment).

**Table 1:** Clinical characteristics of patients and control group

Variable	Anorexic patients (*N* = 25)		Healthy controls (*N* = 25)		*t* value, d*f* = 48	*p* value
	Mean	SD	Mean	SD		
Age	27.1	6.3	24.5	5.2	1.57	0.12
Years of education	16.1	1.4	16.4	0.9	−1.04	0.30
BMI	17.6	2.2	23.4	3.6	−6.77	*0.001*
Duration of illness	9.7	6.6	–	–		
No of previous admissions	3.2	2.7	–	–		
Yale–Brown scale score	20.0	6.2	4.1	5.3	9.49	*0.001*
BDI score	29.8	15.7	6.2	4.1	6.69	*0.001*
EAT-26 scale score	41.9	19.81	4.58	4.48	9.00	*0.001*

The subjects completed the Beck depression inventory (BDI), an instrument for the self-rating of symptoms of depression and the Yale Brown obsessive compulsive scale (Y-BOCS) allowing to evaluate OCD traits or symptoms. Additionally, the eating attitude test (EAT-26) was used to show eating habits and concerns about body weight and shape (Table 1).

### Procedure

Participants attended once to complete clinical assessments and all perceptual tasks.

All assessments were administered in the same time interval.

Study was conducted with Cardiff University Research Ethics Committee (UREC) approval.

### Assessments

The facial expression recognition test (FERT) is a computerised tool based on 25 facial expressions from a standardised series of pictures of facial affect, POFA [8]. Participants view prototypical facial expressions of the six basic emotions (fear, disgust, anger, surprise, happiness, and sadness) and decide the emotion displayed by each facial expression. Emotion labels were visible when participant were making their decisions.

Short recognition memory test for faces (RMTF) is a forced-choice recognition memory test. It consists of 25 unfamiliar grey-scale male faces, which are presented at a rate of one every 3 s. Recognition memory was assessed immediately after the presentation of the stimuli using a two-choice format, each stimulus item being paired with one distracter item [32]. RMTF was used in the study as a control task for the facial expression recognition test (FERT) to assess whether deficits in emotional perception exist independently or are strongly related to impairments in face processing.

The “Reading the Mind in the Eyes” Test (revised version; RME) [4] is a measure of adult “mentalising” and the assessment of how well the participants can put themselves into the mind of another person, and to “tune into” their mental state. In this test, the participant is presented with a series of 36 photographs of the eye region of unknown faces, and is asked to choose which of four words best describe what the person in the photo is thinking or feeling.

The eating attitudes test (EAT-26) is standardised self-report measure of symptoms and concerns characteristic of eating disorders. The tests are rated on a six-point scale (always, usually, often, rarely, sometimes, and never) in response to how often the individual engages in specific behaviours.

### Analysis of data

A power analysis performed using NQuery suggest that a minimum experimental sample size of *N* = 25 would be sufficient to detect the differences in task performance.

Descriptive data are presented as individual values, mean, and standard deviation. Socio-demographic and clinical data and task performance differences between groups were compared using the independent samples *t* test. Pearson correlations were used to investigate correlations between factors. Statistical analyses were performed using SPSS 22.0 statistical software for Microsoft Windows.

## Results

### Demographic data and clinical measures

Socio-demographic and clinical data of participants are included in Table 1.

There were no statistically significant differences between the group with anorexia and healthy controls for age and years of education. However, anorexia group had significantly lower BMI compared to control group (*t* = −6.77; d*f* = 48; *p* < 0.001) and presented with significantly higher level of both OCD (*t* = 9.48; d*f* = 48; *p* < 0.001) and depressive symptoms (*t* = 6.69; d*f* = 48; *p* = *p* < 0.001) (Table 1).

### Social cognition and face recognition

In comparison with controls (using *t* test) individuals with anorexia showed no statistically significant differences in their performance of all facial tests used in the study (Table 2).

**Table 2:** Results of facial tasks in subject groups

	Anorexic patients (*N* = 25)		Healthy controls (*N* = 25)		*t* value, d*f* = 48	*p* value
	Mean	SD	Mean	SD		
FERT	18.4	2.6	19.1	2.3	−0.51	0.61
RMTF	22.8	2.8	23.5	0.8	−0.95	0.34
Eyes test	25.6	5.6	26.7	4.5	−1.25	0.22

### The effect of clinical and demographic variables on task performance

Pearson correlations were performed between mean accuracy scores and clinical variables (e.g. Yale–Brown and BDI, EAT-26, years of education, illness duration and current BMI). Interestingly, in the patient group, there were no statistically significant effect of depression, obsessive–compulsive symptoms, severity of eating problems, chronicity, and current BMI on social cognitive/cognitive tasks performance.

In the patients with anorexia a statistically significant correlation was found between mean score of ‘Reading the mind in the eyes’ test and mean age (*r* = −0.47; *p* < 0.05). In the control group statistically significant correlations were found between mean score of BMI and mean score of RMTF (*r* = −0.60; *p* < 0.01) and additionally between mean age of controls and mean score of RMTF (*r* = −0.42; *p* < 0.05).

The anorexic group was mildly older than healthy controls, but there was no statistically significant difference between groups. Groups were homogeneous in terms of years of education—approximately 16 years of education for both groups included in the research. Highly statistically significant differences between groups were obtained for mean BMI and mean Yale–Brown, BDI and EAT-26 scales scores (all with *p* < 0.001).

## Discussion

Despite significant clinical pathology, the group of individuals with anorexia nervosa did reveal neither deficits in emotion recognition nor in theory of mind compared to the healthy individuals. These results are compatible with those combined by Cardi et al. [6] which claim that participants with EDs did not display specific abnormalities in emotional processing, recognition and empathic response to others’ basic discrete emotions. However, they had poorer facial expressivity and a tendency to turn away from emotional displays. We did not find that individuals with AN had a specific impairment in the recognition of facial expression of emotions, which is in line with some previous studies [2, 17, 20, 21, 33]. Our results correspond with Phillipou and others’ research (2015) that shows participants with AN did not differ from healthy control participants in the areas of attentional focus when viewing the Ekman face stimuli, nor did they differ in emotion identification of these stimuli. Phillipou et al. study encourages idea of intact emotion identification of facial affect stimuli and distinct hyperscanning behaviours when viewing faces in AN [25]. Our service users were similarly accurate in their detection of facial emotion and faces per se relative to the healthy group which stays in line with results of other authors [20]. Harrison et al. [15] in their study examined whether the problems in emotional functioning found in women in acute phase of AN are state or trait effects by comparing women who have already recovered from AN with women with acute AN and female HCs. The results showed that the recovered patients had a significantly higher social and angry-threat attentional bias than HCs, with medium effect sizes, and significantly lower scores on the emotion recognition measure than HCs, with a medium effect size. On the other hand, the recovered group did not significantly differ from the HC group in terms of emotion regulation [15].

Rozenstein et al. [28] have shown that patients with AN are as fast and as accurate in matching both facial identity and facial emotions as their unaffected sisters and unrelated healthy controls.

There have been suggestions that aspects of the anorexia nervosa presentation may be related to autistic type traits [13], and this leads to a hypothesis that impairments in theory of mind might be seen [29]. For that reason participant groups were assessed on the ‘Reading the mind in the eyes’ task, which asked participants to identify a person’s mental state from photographs of people’s eyes. Russell et al. [29] found ToM impairments in the “Reading the Mind in the Eyes task” (RME) in a group of 22 patients with AN. These results were not replicated by Oldershaw et al. [23] and Harrison et al. [14] who did not show any alterations in RME performance in AN patients which stays in line with our data.

Unlike Russell et al. [29], this sample was not impaired on the RME tasks which may stem from sampling differences. Our sample included outpatients with mean BMI equals 17.6, whereas in Russell et al. study, the mean BMI of anorexic inpatient sample was 15.2.

First of all, in the study by Russell et al. [29] there was a statistically significant difference between the AN and HC groups on both mean age and years of education. According to Adenzato et al. [2] both age and education have been proven as crucial variables when assessing ToM by RME whilst previous studies pointed out age and education related effect on RME performance. In our study a statistically significant correlation was found between mean score of ‘Reading the mind in the eyes’ test performed by our patients and their mean age (*p* < 0.05). Furthermore, Adenzato et al. [2] stressed out the potential effect of chronicity on RME performance to test hypothesis proposed by Zucker according to which chronic avoidance of salient social cues in AN may lead to increase dysfunction over time [2]. In our study, the illness duration exceeds 9 years similarly to studies done by Russell et al. [29] and Harrison et al. [14] in which significantly weaker RME performance was found in AN patients. Thus in our study, Zucker’s hypothesis was not supported.

These differences can be explained by concluding that autistic type traits are more pronounced in patients with prolonged starvation and lower BMI. Furthermore, these traits may exacerbate factors that maintain the eating disorder rather than being a causative factor.

Patients with anorexia were significantly more depressed than healthy controls, and showed vivid obsessive–compulsive symptoms compared to controls which remain in line with the previous findings.

Undoubtedly, reliable measurement of social cognition per se remains problematic due to relatively limited psychometric results for many social cognitive tasks used widely in clinical research. As part of the social cognition psychometric evaluation (SCOPE) study, the psychometric properties of eight tasks were assessed by Pinkham et al. [26] based upon the following factors: test–retest reliability, utility as a repeated measure, relationship to functional outcome, practicality and tolerability, sensitivity to group differences, and internal consistency. The widely used in clinical studies the “Reading the Mind in the Eyes task” showed weaker psychometric properties in Pinkham et al. [26].

More longitudinal studies are needed to establish specificity (trait- or state-dependent nature) of the concomitant social cognition deficits in anorexia nervosa using the measures with higher psychometric properties and find out if similar findings are observed in other psychiatric cohorts of patients to tailor subsequent AN management to be more successful and promising in future.

## Conclusions

The findings of the current study corroborate those of Adenzato et al. [2] and Renwick et al. [27] and state that despite significant clinical pathology, the group of individuals with anorexia nervosa did not show any social cognitive/social perception deficits compared to the healthy control group. We did not find that individuals with AN had a specific impairment in the recognition of facial expression of emotions, which is in line with some previous research [17, 20, 21, 33]. This research states that following other studies [11, 27] social perception in people with anorexia nervosa is largely preserved.

Undoubtedly there are not many research projects conducted among community outpatients which is an indisputable strength of our study. This research results stay in line with the study carried out by Morris et al. which states that the acute AN group reported lower levels of empathy than the recovered AN group and HC, but they also reported less antisocial behaviour. No differences were found in emotional recognition or social conformity [22].

Recruitment criteria in our study were highly selective as research included patients with anorexia nervosa (mostly restrictive type) with no concurrent personality disorders diagnosed which made this group strictly homogeneous. Moreover, the study was conducted using well-known, standardised, cross-validated batteries or measures including RMTF as a control task for the FERT.

Cross-sectional design may be a weak point of this particular study, because it did not take the therapy effect into account in long-term. Another limitation of the study is the relatively small sample size. Further longitudinal studies are needed to assess precisely the dynamic changes in emotional processing at various stages of AN, whether they are state- or of trait related.

## Abbreviations

AN: anorexia nervosa
FERT: facial expression recognition test
RMTF: short recognition memory test for faces
RME: reading the mind in the eyes’ test
EAT-26: the eating attitudes test
OCD: obsessive compulsive disorder
Y-BOCS: the Yale–Brown obsessive compulsive scale
BDI: Beck depression inventory
ToM: theory of mind
BMI: body mass index
DSM-5: Diagnostic and Statistical Manual of Mental Disorders, 5th Edition
UK: United Kingdom
ICD-10: International classification of diseases and diagnostic
DSM IV TR: Diagnostic and Statistical Manual of Mental Disorders, 4th Edition, Text Revision
NHS: National Health Service
UREC: Cardiff University Research Ethics Committee
UREC: University Research Ethics Committee
POFA: pictures of facial affect
EDs: eating disorders
SCOPE: social cognition psychometric evaluation
HC: healthy controls

## References

[1] Adolphs R (2001). The neurobiology of social cognition. Curr Opin Neurobiol.

[2] Adenzato M, Todisco P, Ardito RB (2012). Social cognition in anorexia nervosa: evidence of preserved theory of mind and impaired emotional functioning. PLoS One..

[3] Allport GW, Odbert HS (1936). Trait names: a psycho-lexical study. Psychol Monogr.

[4] Baron-Cohen S, Wheelwright S, Hill J, Raste Y (2001). The, “reading the mind in the eyes” test revised version: a study with normal adults, and adults with Asperger syndrome of high- functioning autism. J Child Psychol Psychiatry..

[5] Beadle JN, Paradiso S, Salerno A, McCormick LM (2013). Alexithymia, emotional empathy, and self-regulation in anorexia nervosa. Ann Clin Psychiatry.

[6] Cardi V, Corfield F, Leppanen J, Rhind C, Deriziotis S, Hadjimichalis A, Treasure J (2015). Emotional processing, recognition, empathy and evoked facial expression in eating disorders: an experimental study to map deficits in social cognition. PLoS One.

[7] Dmitrzak-Weglarz M, Jaracz J, Slopien A, Maciukiewicz M, Rajewski A (2010). Emotion recognition deficits in adolescent patients with anorexia nervosa. Neuropsychiatria i Neuropsychologia.

[8] Ekman P, Friesen WV (1976). Measuring facial movement. Environ Psychol Nonverbal Behav..

[9] Fairburn CG, Shafran R, Cooper Z (1999). A cognitive behavioural theory of anorexia nervosa. Behav Res Ther..

[10] Fonagy P. Affect regulation, mentalization, and the development of the self. 1st edn. New York: Other Press LLC; 2002.

[11] Goddard E, Carral-Fernández L, Denneny E, Campbell IC, Treasure J (2014). Cognitive flexibility, central coherence and social emotional processing in males with an eating disorder. World J Biol Psychiatry..

[12] Hamaker EL, Nesselroade JR, Molenaar PCM (2007). The integrated trait–state model. J Res Pers.

[13] Hambrook D, Tchanturia K, Schmidt U, Russel T, Treasure J (2008). Empathy, systemizing and autistic traits in anorexia nervosa: a pilot study. Br J Clin Psychol..

[14] Harrison A, Sullivan S, Tchanturia K (2009). Treasure. Emotion recognition and regulation in anorexia nervosa. J Clin Psychol Psychother..

[15] Harrison A, Tchanturia K, Treasure J (2010). Attentional bias emotion recognition and emotion regulation in anorexia state or trait. Biol Psychiatry..

[16] Jänsch C, Harmer C, Cooper MJ (2009). Emotional processing in women with anorexia nervosa and in healthy volunteers. Eat Behav.

[17] Kessler H (2006). Alexithymia and facial emotion recognition in patients with eating disorders. Int J Eat Disord..

[18] Kucharska-Pietura K, Mortimer A, Tylec A (2012). Social cognition and visual perception in schizophrenia inpatients treated with first-and second-generation antipsychotic drugs. Clin Schizophr Relat Psychoses..

[19] Krug I, Penelo E, Fernandez-Aranda F, Anderluh M, Bellodi L (2013). Alexithymia and facial emotion recognition in patients with eating disorders control study. J Health Psychol..

[20] Medina-Pradas C (2012). Emotional theory of mind in eating disorders. Int J Clin Health Psycol..

[21] Mendlewicz L (2005). Decoding emotional facial expressions in depressed and anorexic patients. J Affect Disord..

[22] Morris R, Bramham J, Smith E, Tchanturia K (2014). Empathy and social functioning in anorexia nervosa before and after recovery. Cognitive Neuropsychiatry.

[23] Oldershaw A, DeJong H, Hambrook D, Broadbent H, Tchanturia K, Treasure J, Schmidt U (2012). Emotional processing following recovery from anorexia nervosa. Eur Eat Disord. Rev..

[24] Oldershaw A, Hambrook D, Stahl D, Tchanturia K, Treasure J, Schmidt U (2011). The socio-emotional processing stream in anorexia nervosa. Neurosci Biobehav Rev..

[25] Phillipou A, Abel LA, Castle DJ, Hughes ME, Gurvich C, Nibbs RG, Rossell SL (2015). Self perception and facial emotion perception of others in anorexia nervosa. Front Psychol.

[26] Pinkham AE, Penn DL, Green MF, Harvey PD (2016). Social cognition psychometric evaluation: results of the initial psychometric study. Schizophr Bull.

[27] Renwick B, Dejong H, Kenyon M, Samarawickrema N, Loomes R, Watson C, Schmidt U (2013). Social perception in people with eating disorders. European Psychiatry.

[28] Rozenstein MH, Latzer Y, Stein D, Eviatar Z (2011). Perception of emotion and bilateral advantage in women with eating disorders, their healthy sisters, and nonrelated healthy controls. J Affect Disord..

[29] Russell TA, Schmidt U, Doherty L, Young V, Tchanturia K (2009). Aspects of social cognition in anorexia nervosa: affective and cognitive theory of mind. Psychiatry Res..

[30] Spielberger CD, Sydeman SJ, Maruish ME (1994). State–trait anxiety inventory and state–trait anger expression inventory. The use of psychological testing for treatment planning and outcome assessment.

[31] Tapajóz Pereira de Sampaio F, Soneira S, Aulicino A, Harris P, Allegri RF. Emotional reactivity to social stimuli in patients with eating disorders. Psychiatry Res. 2015. doi: 10.1016/j.psychres.2015.07.049. 10.1016/j.psychres.2015.07.049 26257086

[32] Warrington EK (1996). The Camden memory tests manual.

[33] Zonnevylle-Bender MJ (2004). Emotional functioning in anorexia nervosa patients: adolescents compared to adults. Depress Anxiety..

